# Ethyl Pyruvate Stimulates Regulatory T Cells and Ameliorates Type 1 Diabetes Development in Mice

**DOI:** 10.3389/fimmu.2018.03130

**Published:** 2019-01-10

**Authors:** Ivan Koprivica, Milica Vujičić, Dragica Gajić, Tamara Saksida, Ivana Stojanović

**Affiliations:** Department of Immunology, Institute for Biological Research “Siniša Stanković”, University of Belgrade, Belgrade, Serbia

**Keywords:** regulatory T cells (Treg), type 1 diabetes (T1D), ethyl pyruvate, inflammation, immunoregulation

## Abstract

Type 1 diabetes (T1D) is an autoimmune disease in which a strong inflammatory response causes the death of insulin-producing pancreatic β-cells, while inefficient regulatory mechanisms allow that response to become chronic. Ethyl pyruvate (EP), a stable pyruvate derivate and certified inhibitor of an alarmin–high mobility group box 1 (HMGB1), exerts anti-oxidant and anti-inflammatory properties in animal models of rheumatoid arthritis and encephalomyelitis. To test its therapeutic potential in T1D, EP was administered intraperitoneally to C57BL/6 mice with multiple low-dose streptozotocin (MLDS)-induced T1D. EP treatment decreased T1D incidence, reduced the infiltration of cells into the pancreatic islets and preserved β-cell function. Apart from reducing HMGB1 expression, EP treatment successfully interfered with the inflammatory response within the local pancreatic lymph nodes and in the pancreas. Its effect was restricted to boosting the regulatory arm of the immune response through up-regulation of tolerogenic dendritic cells (CD11c^+^CD11b^−^CD103^+^) within the pancreatic infiltrates and through the enhancement of regulatory T cell (Treg) levels (CD4^+^CD25^high^FoxP3^+^). These EP-stimulated Treg displayed enhanced suppressive capacity reflected in increased levels of CTLA-4, secreted TGF-β, and IL-10 and in the more efficient inhibition of effector T cell proliferation compared to Treg from diabetic animals. Higher levels of Treg were a result of increased differentiation and proliferation (Ki67^+^ cells), but also of the heightened potency for migration due to increased expression of adhesion molecules (CD11a and CD62L) and CXCR3 chemokine receptor. Treg isolated from EP-treated mice had the activated phenotype and T-bet expression more frequently, suggesting that they readily suppressed IFN-γ-producing cells. The effect of EP on Treg was also reproduced *in vitro*. Overall, our results show that EP treatment reduced T1D incidence in C57BL/6 mice predominantly by enhancing Treg differentiation, proliferation, their suppressive capacity, and recruitment into the pancreas.

## Introduction

Type 1 diabetes (T1D) is an autoimmune disease that develops as a consequence of β-cell death and subsequent lack of insulin. The cells that infiltrate the pancreatic islets and destroy β-cells are usually M1 pro-inflammatory macrophages and dendritic cells (DC) that present autoantigens to CD8^+^ cytotoxic lymphocytes, IFN-γ-producing Th1 and IL-17-producing Th17 lymphocytes. The opposing components of the immune response are M2 anti-inflammatory macrophages, as well as tolerogenic DC that enable activation of regulatory T lymphocytes (Treg) (1). The balance between pro-inflammatory and regulatory cells is a common target for new experimental T1D therapeutics, for example, ethyl pyruvate (EP). This is a stable pyruvate derivative that possesses anti-oxidant and anti-inflammatory properties. It is a genuine inhibitor of high mobility group box 1 (HMGB1), a protein that, like histones, binds DNA and regulates transcription (2). More importantly, HMGB1 can be secreted by monocytes, macrophages and dendritic cells and can act as an alarmin to promote inflammation and generation of reactive oxygen species (ROS) and reactive nitrogen intermediates (RNI) (3, 4). Either through inhibition of HMGB1 or interaction with other still unknown molecules, EP has been found beneficial in the treatment of various inflammatory disorders, including myocardial ischemia-reperfusion injury (5), sepsis (6), pancreatitis (7), colitis (8), inflammatory arthritis (9), and experimental autoimmune encephalomyelitis (10). Thorough analysis of EP's mechanism of action in the autoimmune process that targets CNS suggests that EP ameliorated the symptoms of experimental autoimmune encephalomyelitis through inhibition of HMGB1 secretion from microglia/macrophages (10, 11). The activity of phagocytic cells (ROS production and antigen presentation properties) and Th cells (IFN-γ and IL-17 production) was significantly impaired by the action of EP (10, 11). The effects of EP include more than only the inhibition of the pro-inflammatory response; EP also boosts the regulatory component of immunity, as recent findings indicate that EP promotes the differentiation of tolerogenic DC (tolDC) *in vitro* (unpublished data). However, there are no data on the possible effect of EP on Treg.

So far, EP has been mostly used to treat the secondary effects that diabetes and the resulting hyperglycemia have on the retina (12), kidneys (13), or liver (14). Having in mind that HMGB1 enhances the progression of T1D in NOD mice (15), the application of EP might prove beneficial for the treatment of T1D.

## Material and Methods

### Animals

C57BL/6 mice were kept at the animal facility at the Institute for Biological Research “Sinisa Stankovic,” under standard conditions with free access to food and tap water. All experimental procedures were approved by the Ethic Committee at the Institute for Biological Research “Sinisa Stankovic” (App. No 01-11/17 - 01-2475) in accordance with the Directive 2010/63/EU.

### T1D Induction and EP Treatment

T1D was induced in 2 months old male C57BL/6 mice using multiple low doses of streptozotocin (MLDS) that were given intraperitoneally for 5 consecutive days. Streptozotocin (STZ) (40 mg/kg bw, Sigma-Aldrich, St. Louis, MO, USA) was dissolved in cold 0.1 M citrate buffer (pH 6) just prior to administration. Ethyl pyruvate (EP) (100 mg/kg bw, Sigma-Aldrich) was dissolved in Hartmann's solution (Hemofarm A.D., Vršac, Serbia) and administered intraperitoneally, starting from the first dose of STZ for 9 days in total. STZ and EP injections were given 3 h apart. MLDS-treated group also received the diluent in equal volume. Mice were monitored for the development of hyperglycemia by weekly measurements of blood glucose levels, using a glucometer (Sensimac, IMACO GmbH, Lüdersdorf, Germany). Animals were considered hyperglycemic if their blood glucose level was higher than 11 mmol/l in non-fasted animals. The presence of ketones in urine (as an indirect measure of high glucose levels) was detected by URiSCAN test strips (YD Diagnostics, Kyunggi-Do, South Korea). *Ex vivo* analyses were performed between the 11th and the 15th day after the first STZ injection.

### Histological Analysis

To assess the incidence of inflammatory changes and the degree of islet cell destruction, pancreata were collected on the 35th day after the first STZ injection, embedded in paraffin, sectioned (5 μm thick sections, at least 200 μm between sections), stained with Mayer's hematoxylin (Bio-Optica, Milan, Italy) and examined by light microscopy (Zeiss Imager Z1, AXIO, Carl Zeiss Meditec AG, Oberkochen, Germany). Insulitis scoring was performed by examining at least 20 islets per pancreas and graded in a blinded fashion as follows: grade 0, intact islet; grade 1, peri-islet infiltrate; grade 2, heavy intra-islet infiltrate. Results are expressed as a percentage of graded islets out of the total number of islets.

Immunohistochemical staining was performed on pancreatic sections collected on the 15th day after the first STZ injection in the following manner. To assess the presence of insulin, PE conjugated rabbit anti-mouse insulin antibody (1:400, Cell Signaling Technology, Danvers, MA, USA) was used, while counterstaining was carried out with Hoechst 33342 dye (2 μl/ml, ChemoMetec, Allerød, Denmark). For the negative control, rabbit anti-goat IgG-biotin (Vector Laboratories, Burlingame, CA, USA) coupled with streptavidin-PE (ThermoFisher Scientific, Waltham, MA, USA) was used. Image acquisition (20×) was performed using an AxioVision microscope (Carl Zeiss Meditec AG, Germany). The presence of insulin in the pancreatic islets was determined with Fiji, an open-source distribution of ImageJ software for biological image analysis (16). The acquired images were converted to gray scale, and fluorescence intensity was quantified by measuring the mean gray value, which represents the sum of gray values of all pixels in the selection divided by the number of pixels. For HMGB1 assessment, anti-mouse HMGB1 antibody (1:500, Invitrogen, Carlsbad, CA, USA) was paired with a secondary HRP conjugated anti-mouse IgG (1:1,000, Invitrogen), incubated with ExtrAvidin peroxidase (1:10, Sigma-Aldrich) and finally stained with DAB chromogen solution (1:50, DakoCytomation, Glostrup, Denmark), while counterstaining was carried out with hematoxylin. For the negative control, HRP conjugated anti-mouse IgG-treated sections were used. The presence of HMGB1 in pancreatic islets was measured in the following way. Pancreatic islets were photographed at the magnification of 20×, and HMGB1 positive regions were determined using the Color Picker Threshold Plugin within Icy, open-source bioimage processing software (17). Using a representative pancreatic section, at least 15 positive colors (shades of darker brown) and 15 negative colors (blue, white and light brown) were selected as standard recognition patterns. Those were then applied to all pancreatic sections to differentiate between stained and unstained tissue regions. The presence of HMGB1 in pancreatic islets was expressed as a relative percentage of the HMGB1-positive islet area divided by the area of the whole pancreatic islet.

### Cell Isolation

Spleen and pancreatic lymph node (PLN) cells were obtained by passing the tissue through the cell strainer (40 μm) and after removal of the erythrocytes by RBC Lysis Buffer (eBioscience, San Diego, CA, USA). Pancreatic infiltrates were obtained by collagenase type V (Sigma-Aldrich) digestion of the pancreatic tissue (from which lymph nodes were previously removed). Tissue was cut into small pieces and shaken for 10 min at 37°C. Digests were passed through a 20 μm cell strainer and washed several times in Hank's balanced salt solution. The cells were finally resuspended in RPMI 1640 supplemented with 5% fetal calf serum (FCS), 1% penicillin and streptomycin (all from PAA Laboratories, Pasching, Austria), 2 mM L-glutamine and 25 mM HEPES. CD4^+^CD25^−^, CD25^+^ or CD25^−^ cells were marked for separation from the spleen, cervical and mesenteric lymph node cell suspension by incubation with biotin conjugated anti-mouse CD4 (1:60) or biotin conjugated anti-mouse CD25 (1:100) antibodies (both from eBioscience). They were then resuspended in cold magnetic bead buffer (PBS+0.5% BSA+2 mM EDTA) containing BD IMag™ Streptavidin Particles Plus–DM (1:20, BD Biosciences, Bedford, MA, USA). The appropriate cells were purified by placement in a BD IMag™ Cell Separation Magnet (BD Biosciences), 3× for 8 min, and finally resuspended in a T lymphocyte medium–RPMI 1640 supplemented with 10% FCS, 1% penicillin and streptomycin, 0.02 mM Na-pyruvate, 5 μM β-mercaptoethanol, 2 mM L-glutamine, and 25 mM HEPES.

### Detection of Extracellular and Intracellular Markers by Flow Cytometry

Surface molecules were detected on viable cells dispersed in PBS+1% BSA. The following antibodies were used: anti-mouse CD4 PerCP-Cyanine5.5 (rat IgG2a,κ), anti-mouse CD4-FITC (rat IgG2b,κ), CD4-APC (rat IgG2b,κ), anti-mouse CD8-PE (rat IgG2a,κ), anti-mouse B220-FITC (rat IgG2a,κ), anti-mouse CD19-PE-Cy5 (rat IgG2a,κ), CD11c-PE-Cy5 (Armenian hamster IgG), F4/80-FITC (rat IgG2a,κ), CD25-Alexa Fluor® 488 (rat IgG1,λ), CD25-PE (rat IgG1,λ), CD101-PE (rat IgG2a,κ), CD62L-PE-Cy7 (rat IgG2a,κ), CD206-PE (rat IgG2b,κ), CD127 APC-eFluor 780 (rat IgG2a,κ), MHC II-FITC (rat IgG2b,κ), CD357 (GITR)-FITC (rat IgG2b,κ), CD80-PE-Cy5 (Armenian hamster IgG), CD86-PE-Cy5 (rat IgG2a,κ), CD103-FITC (Armenian hamster IgG), CD5-FITC (rat IgG2a,κ), CD11a-FITC (rat IgG2a,κ), CD40-PE (Armenian hamster IgM,κ), anti-rat IgG-PE (all from ThermoFisher Scientific), CD11b (rat IgG2b,κ) (BD Biosciences), PD-1 (rat IgG2a,κ) (Abcam, Cambridge, MA, USA). The staining was performed for 30 min at 4°C. Regulatory T cells (Treg) were detected by Mouse Regulatory T cell Staining Kit (FoxP3) according to the manufacturer's instructions (eBioscience). For intracellular cytokine staining, cells were stimulated with Cell Stimulation Cocktail (plus protein transport inhibitors) (1:500, eBioscience) for 4 h. Cells were fixed in 2% paraformaldehyde, permeabilized and stained with the following antibodies: anti-mouse IFN-γ-PE (rat IgG1,κ), IL-4-PE (rat IgG1,κ) (both from eBioscience) or IL-17-PE (rat IgG1,κ) (BD Biosciences). Isotype-matched controls were included in all experiments (eBioscience). For Ki67-FITC (goat polyclonal antibody) (SantaCruz Biotechnology, San Diego, USA), T-bet-PE (mouse IgG1,κ) and RORγT-PE (rat IgG1,κ) (eBioscience) antibodies, cells were permeabilized using the same protocol as for FoxP3 (Treg) detection. Cells were acquired on Partec CyFlow Space by FlowMax software (Partec, Görlitz, Germany) or by FACSCalibur and BD FACSAria III (BD Biosciences) and analyzed by FlowMax (Partec) or FlowJo software (Treestar, Ashland, OR, USA). Cells were first gated on live cells (empirically determined) and then further gated appropriately to the required analysis.

### Measurement of Intracellular Nitric Oxide and Reactive Oxygen Species

4-Amino-5-methylamino-2′7′-difluoro-fluorescein diacetate (DAF-FM diacetate; Molecular Probes, Leiden, The Netherlands) was used as an indicator of intracellular nitric oxide (NO). Briefly, pancreatic infiltrates were incubated with 2 μM DAF-FM diacetate for 1 h at 37°C, washed, and then incubated for 15 min at 37°C in phenol red- and serum-free RPMI-1640 for the completion of de-esterification of intracellular diacetates. The cells were than washed and resuspended in PBS. Green (FL1) fluorescence emission was measured with Partec CyFlow Space and analyzed by FlowMax software. Dihydrorhodamine 123 (DHR) was used to reactive oxygen species (ROS). After isolation, cells were immediately exposed to DHR (5 μM) for 20 min at 37°C. After being washed, the cells were analyzed with the flow cytometer. The mean fluorescence intensity (MFI) as a measure of intracellular production of NO and ROS was measured in the macrophage gate (higher SSC level compared to the lymphocyte gate).

### Immunoblot

*Ex vivo* isolated cells (5 × 10^6^) from PLN or pancreatic infiltrates, purified CD25^+^ cells (5 × 10^5^) or *in vitro* cultured CD4^+^CD25^−^ (5 × 10^6^) were lysed in the buffer containing 62.5 mmol/l Tris–HCl (pH 6.8 at RT), 2% SDS, 10% glycerol, 50 mmol/l DTT, 0.01% bromophenol blue (all from Sigma-Aldrich). All samples were subjected to electrophoresis on 12% SDS–polyacrylamide gel (SDS–PAGE). After electro-transferring the samples to polyvinylidene difluoride membranes at 5 mA/cm2, using a semi-dry blotting system (Semi-Dry Transfer Unit, GE Healthcare, Buckinghamshire, England), the membranes were blocked with PBST (PBS+0.1% Tween-20, Sigma-Aldrich)+5% BSA and probed with specific antibodies dissolved in PBST+1% BSA. Secondary antibodies for anti-mouse CTLA-4 (l:1,000, Invitrogen) were FITC conjugated anti-armenian and syrian hamster IgG cocktail (1:1,000, BD Biosciences) and HRP conjugated anti-mouse IgG (1:5,000, Invitrogen), for anti-mouse IL-10 (l:600, eBioscience) or anti-mouse IL-2 (l:500, eBioscience) it was HRP conjugated anti-rat IgG (1:5,000, eBioscience), and for anti-mouse HMGB1 (1:1,500, Invitrogen) or anti-mouse β-actin (1:1,000, Sigma-Aldrich) it was HRP conjugated anti-mouse IgG (1:5,000, Invitrogen). Detection was performed by using Immobilon Western Chemiluminescent HRP Substrate (Millipore, Billerica, MA, USA), while photographs were made by X-ray films (Kodak, Rochester, NY, USA). Densitometry was performed with Scion Image Alpha 4.0.3.2 (Scion Corporation, Frederick, MD, USA), and the production of specific proteins was expressed relative to the production of β-actin.

### *In vitro* Suppression Assay and Transmigration Assay

For the suppression assay, CD4^+^CD25^−^ cells isolated from either EP-treated or diabetic animals were first incubated with 2 μM CFSE (Invitrogen) for 20 min at RT and 5 min at 37°C, washed and resuspended in the T lymphocyte medium. A U-bottom 96-well plate (Sardstedt, Numbrecht, Germany) was coated with anti-mouse CD3 (1 μg/ml, eBioscience), and an equal number of purified CD4^+^CD25^−^ cells (25 × 10^3^) was placed in each well. CD25^+^ cells were then added in a series of dilutions, starting from 25 × 10^3^ cells per well, and continuing to a 2×, 4×, 8×, and 16× lesser number of cells. Certain wells contained only CD4^+^CD25^−^ cells, as control. The cells were also stimulated by the addition of anti-mouse CD28 (1 μg/ml, eBioscience) to the T lymphocyte medium. After 3 days of cultivation, the cells were washed, resuspended in PBS and analyzed by flow cytometry.

For the transmigration assay, a special chemotaxis system was used (ChemoTX System, Neuro Probe Inc., MD, USA). CD25^+^ or CD25^−^ cells isolated from either EP-treated or diabetic animals (10^5^ cells in 50 μl) were placed above a membrane and their migratory abilities were tested, either toward isolated pancreatic islets [isolation described in 18], medium containing CXCL12 (10 ng/ml, Gibco, ThermoFisher Scientific), or plain medium as control. After 4 h, the number of cells that migrated through the membrane and into the wells was determined by LUNA-II™ Automated Cell Counter (Logos Biosystems, Gyeonggi-do, South Korea).

### *In vitro* Th Differentiation Assay

CD4^+^CD25^−^ cells (5 × 10^6^) isolated from untreated healthy animals were stimulated in an adequate manner for the purpose of Th cell differentiation. All cells received stimuli from plate-bound anti-CD3 (1 μg/ml) and soluble anti-CD28 antibodies (1 μg/ml) (both from eBioscience). For Th1 differentiation the cells were additionally stimulated with IL-12 (20 ng/ml, R&D Systems, Minneapolis, MN, USA) and anti-IL-4 antibody (10 ng/ml, eBioscience), for Th17 differentiation the cells were stimulated with TGF-β (10 ng/ml, R&D Systems) and IL-6 (10 ng/ml, R&D Systems), while for Treg differentiation the cells were stimulated with either TGF-β (2 ng/ml) and IL-2 (10 ng/ml) (both from R&D Systems) (complete stimulation), or only with IL-2 (incomplete stimulation). EP (125 μM) was administered 24 h after the beginning of the culture, and after 4 more days of cultivation the cells were analyzed for the presence of certain Th subsets by flow cytometry as described above.

### ELISA

Splenocytes were cultured in the presence of LPS (100 ng/ml) for 48 h and cytokine concentration in cell culture supernatants was determined by sandwich ELISA using MaxiSorp plates (Nunck, Rochild, Denmark) and anti-mouse paired antibodies according to the manufacturer's instructions. Supernatants from previously described *in vitro* differentiation experiments were also collected after 5 days of cultivation. Samples were analyzed in duplicate for murine TNF, IL-1β, and TGF-β (eBioscience) and absorbance was measured by LKB microplate reader (LKB Instruments, Vienna, Austria) at 450 and 570 nm. A standard curve created from the known concentrations of appropriate recombinant cytokines was used to calculate concentration values of measured cytokines.

### Statistical Analysis

Data are presented as mean ± SD. The presented results are representative of four repeated experiments with comparable results. The significance of differences between groups was determined by two-tailed Student's *t*-test. In addition, for the analysis of the results with considerable deviations a Mann-Whitney non-parametric test was used since it produced more stringent p. The usage of this test is specified in the appropriate figure legends. Differences are regarded as statistically significant if *p* < 0.05. Statistical analyses were performed using GraphPad Prism 5 software (GraphPad Software, Inc., La Jolla, CA, USA).

## Results

### The Effect of EP on T1D Development

**Figure 1:**
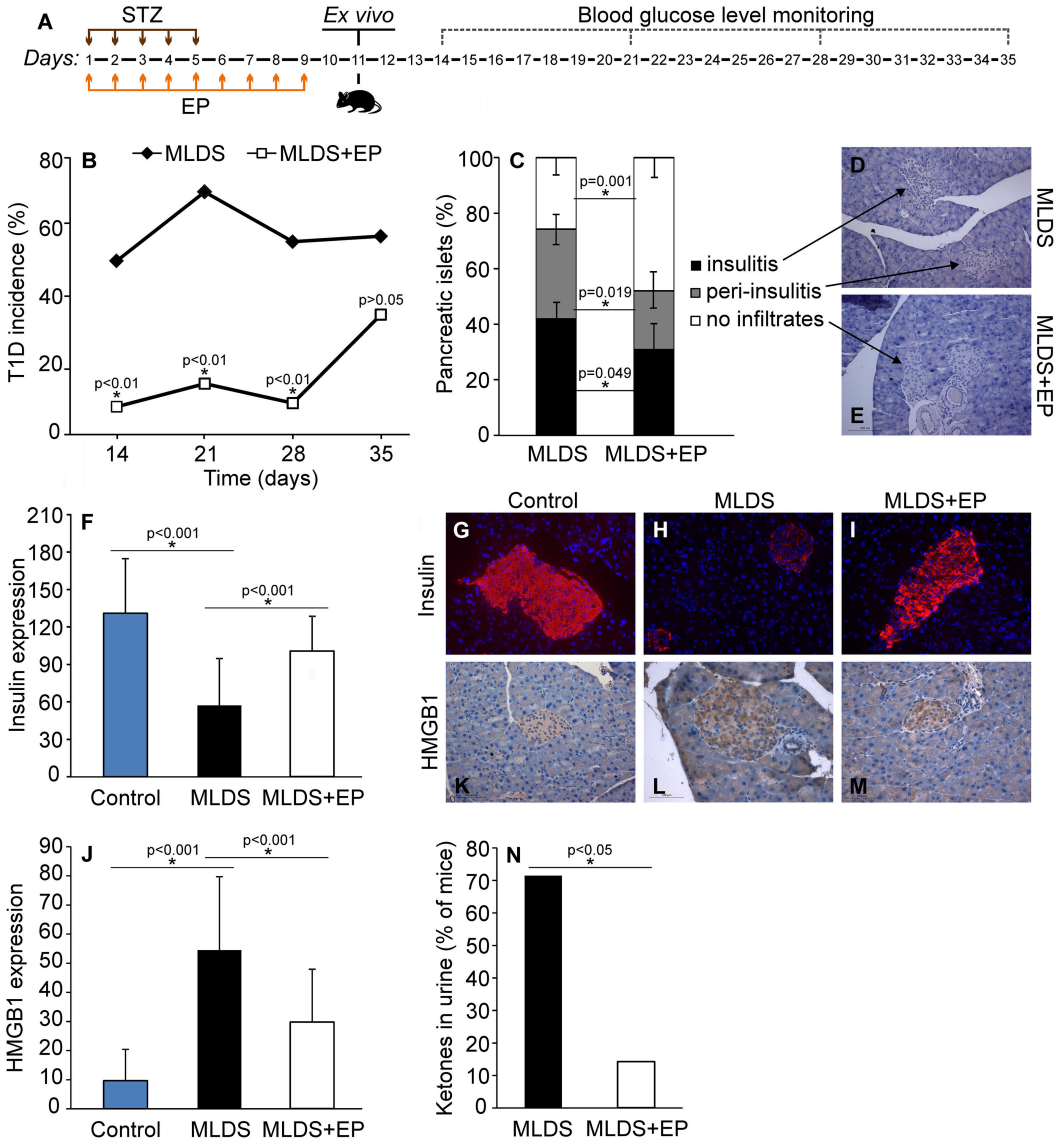
The effect of EP on T1D development. **(A)** Diagram of diabetes induction, EP treatment and diabetes monitoring by weekly measurements of blood glucose levels. **(B)** T1D incidence presented as a proportion of C57BL/6 mice with glycemia higher than 11 mmol/L. **(C)** The proportion of pancreatic islets without immune cell infiltrates, with infiltrates surrounding the islets (peri-insulitis) and with infiltrates within the islet (insulitis). Representative images of pancreatic islets from MLDS-treated mice **(D)** or EP-treated mice **(E)**, stained with hematoxylin. **(F)** Insulin expression was determined by analyzing fluorescence intensity with Fiji software. Representative images of pancreatic islets from a control healthy animal **(G)**, MLDS-treated mice **(H)** or EP-treated mice **(I)** stained for insulin visualization (red) and with Hoechst 33342 (nucleus – blue). **(J)** HMGB1 expression within pancreatic β-cells was determined using the Color Picker Threshold Plugin within Icy software. Representative images of pancreatic islets from a control healthy animal **(K)**, MLDS-treated mice **(L)** or EP-treated mice **(M)** stained for HMGB1. **(N)** The proportion of mice with ketones in urine. All groups consisted of 7-10 animals. *p < 0.05 represents a statistically significant difference between MLDS+EP-treated compared to MLDS-treated mice.

In order to estimate the effect of EP on T1D development in C57BL/6 mice, EP was applied prophylactically for 9 days starting from the first dose of STZ (Figure 1A). In contrast to the control mice that developed hyperglycemia on the 14th day, mice treated with EP had a significantly lower incidence of diabetes (Figure 1B). Also, the number of islets with mononuclear infiltrates (counted on the 35th day from the beginning of the experiment) was lower in EP-treated mice compared to diabetic mice (Figures 1C–E), while the presence of functional insulin^+^ β-cells was higher and similar to that in healthy islets (Figures 1F–I). Isotype staining for insulin is presented in Figure S1A. Since there is evidence of higher expression of HMGB1 in β-cells of NOD mice that develop spontaneous T1D (15), we measured the expression of HMGB1 in the β-cells of mice treated with MLDS. Compared to healthy, untreated mice, HMGB1 expression in diabetic mice was significantly higher, and EP-treatment successfully suppressed the observed change (Figures 1J–M). Background staining is presented in Figure S1B. In addition, throughout the examination period mice treated with EP gained weight normally (data not shown) and only 14% of EP-treated mice had ketones in urine compared to 71% of diabetic ones (Figure 1N).

### The Influence of EP Treatment on Innate Antigen-Presenting Cells

**Figure 2:**
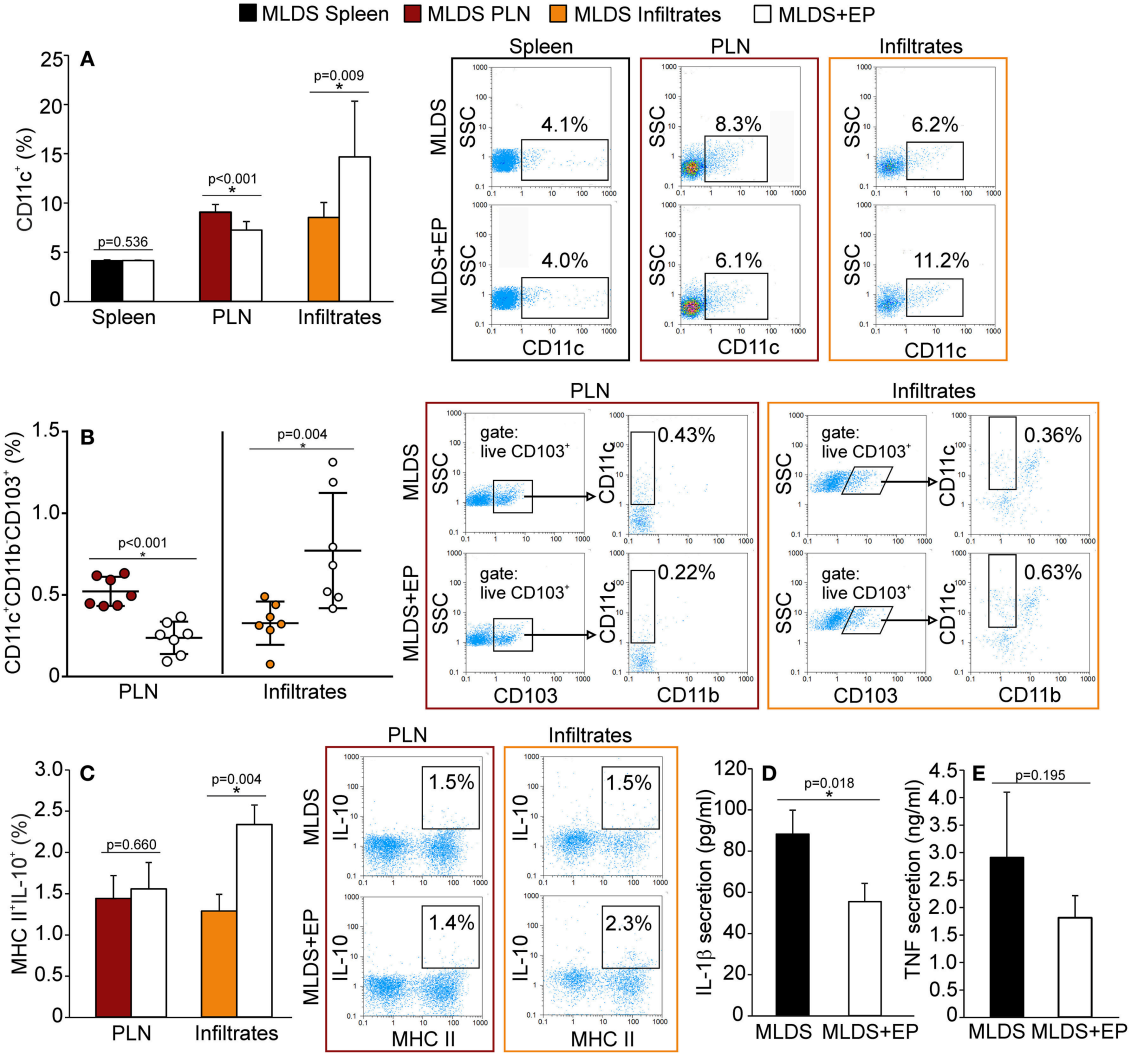
The influence of EP on innate antigen-presenting cells. The proportion of all cells, isolated from the spleen, pancreatic lymph nodes (PLN) or pancreatic infiltrates, was measured by flow cytometry. **(A)** The proportion of CD11c^+^ dendritic cells, along with representative dot plots. **(B)** The proportion of tolerogenic DC (CD11c^+^CD11b^−^CD103^+^), along with representative dot plots (first gated on live CD103^+^ cells, followed by the gate on CD11c^+^CD11b^−^). Statistical analysis for CD11c^+^ and tolerogenic DC was performed by Mann-Whitney non-parametric test. **(C)** Proportion of IL-10^+^ cells within MHC II^+^ population, along with representative dot plots. Secretion of IL-1β **(D)** and TNF **(E)** from LPS-treated splenocytes cultured *ex vivo* for 48 h and measured by ELISA. All measurements were performed on samples from at least 7 animals per group. *p < 0.05 represents a statistically significant difference between cells of MLDS+EP compared to those of MLDS-treated mice.

*Ex vivo* analysis of cells from spleen, PLN and pancreatic infiltrates showed that EP treatment did not change the number of F4/80^+^ macrophages (Figure S2A), while it significantly affected the proportion of dendritic cells (Figure 2A). More precisely, the proportion of CD11c^+^ DC was significantly down-regulated in PLN, while their number was simultaneously increased within the pancreatic infiltrates (Figure 2A).

To explore whether EP changed the ratio between pro-inflammatory M1 and anti-inflammatory M2 macrophage subsets, we detected F4/80^+^CD40^+^ M1 and F4/80^+^CD206^+^ M2 macrophages in the pancreatic infiltrates. The results indicate that EP had no impact on either of the examined macrophage subsets (Figure S2B). However, it did increase the proportion of CD11c^+^CD11b^−^CD103^+^ tolerogenic DC in the pancreatic infiltrates (Figure 2B). These cells seemed to migrate from PLN to the pancreas, since their number was reduced in the nodes after EP treatment (Figure 2B).

To examine the function of antigen-presenting cells, we measured their ability to produce NO and ROS. Despite being an anti-oxidant, EP did not affect the production of NO or ROS in phagocytic cells within the pancreas (Figure S2C). Also, EP did not influence cells' antigen presentation capacity since the expression of co-stimulatory molecules CD80 and CD86 remained the same on MHC class II^+^ cells after EP treatment (Figure S2D). However, EP significantly stimulated the suppressive function of MHC class II^+^ cells since the production of IL-10 in these cells was up-regulated (Figure 2C). Accordingly, when analyzing LPS-stimulated splenocytes, we found that cells isolated from EP-treated mice produced lower amounts of IL-1β (Figure 2D) compared to cells isolated from diabetic mice, while the secretion of TNF was similar (Figure 2E).

### The Effect of EP on Adaptive Immunity in Diabetic Mice

**Figure 3:**
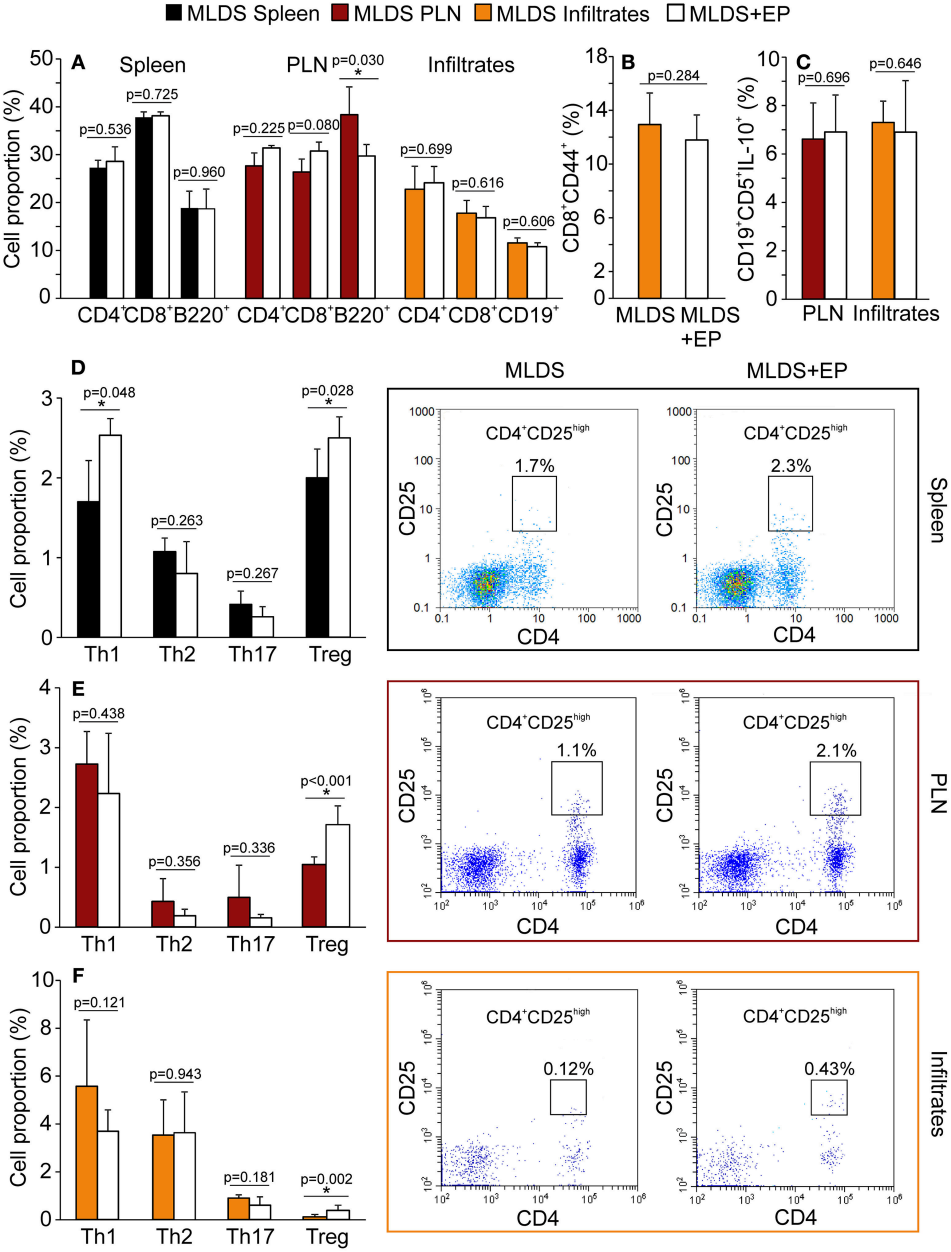
Phenotypic analysis of adaptive immune cells after EP treatment. All cell proportions were measured by flow cytometry. **(A)** The proportion of Th (CD4^+^), cytotoxic lymphocytes (CD8^+^) or B lymphocytes (B220^+^ or CD19^+^) in spleen, PLN or pancreatic infiltrates. **(B)** The proportion of activated cytotoxic lymphocytes (CD8^+^CD44^+^) in the pancreatic infiltrates. **(C)** The proportion of regulatory B cells (CD19^+^CD5^+^IL-10^+^) within PLN and pancreatic infiltrates. The proportion of Th subsets: Th1 (CD4^+^IFN-γ^+^), Th2 (CD4^+^IL-4^+^), Th17 (CD4^+^IL-17^+^) and Treg (CD4^+^CD25^high^) within the spleen **(D)**, PLN **(E)**, and pancreatic infiltrates **(F)** of MLDS or MLDS+EP-treated mice. Representative dot plots for CD4^+^CD25^high^ on the right hand side. All measurements were performed on samples from 7 animals per group. *p < 0.05 represents a statistically significant difference between cells of MLDS+EP compared to those of MLDS-treated mice.

Treatment with EP did not significantly change the proportion of CD4^+^, CD8^+^ or B lymphocytes in the spleen, PLN or pancreatic infiltrates, except for the reduction in B lymphocytes in PLN (Figure 3A). Further insight into CD8^+^ activity revealed that these cells were similarly activated in both diabetic and EP-treated mice (Figure 3B). As for B lymphocytes, the proportion of their regulatory subset (Breg, CD19^+^CD5^+^IL-10^+^) was the same between the groups both in PLN and in the pancreatic infiltrates (Figure 3C). All gating strategies and representative dot plots are shown in Figures S3A–F. After examining the Th subsets (Figures 3D–F), we found that the proportion of CD4^+^CD25^high^ cells (Treg) was up-regulated after EP treatment in all tested compartments. Since the most convincing effect of EP was exerted on Treg, further analysis was focused on Treg function within the local pancreatic environment. Gating strategies for Th1, Th2, and Th17 and representative dot plots are shown in Figures S4A–C.

### *In vivo* EP Effect on Treg Function

**Figure 4:**
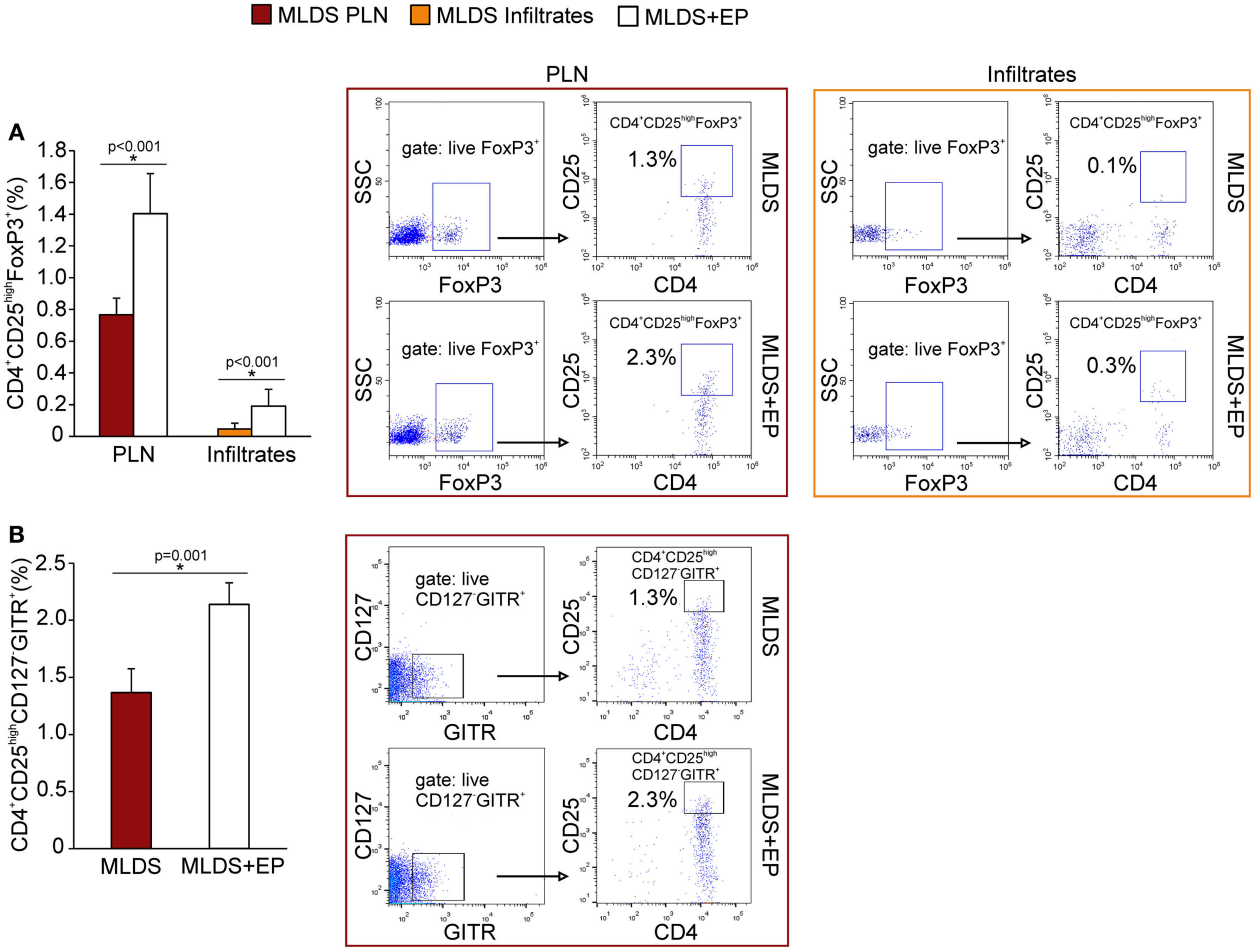
Phenotypical characterization of Treg after EP treatment. **(A)** Proportion of Treg (CD4^+^CD25^high^FoxP3^+^) within PLN and pancreatic infiltrates, along with representative dot plots (first gated on live FoxP3^+^ cells, followed by the gate on CD4^+^CD25^high^). **(B)** The proportion of Treg within PLN that express GITR and are negative for CD127, along with the representative dot plots (first gated on live CD127^−^GITR^+^ cells, followed by the gate on CD4^+^CD25^high^). All measurements were performed on samples from at least 7 animals per group. Statistical analysis was performed by Mann-Whitney non-parametric test. *p < 0.05 represents a statistically significant difference between cells of MLDS+EP compared to those of MLDS-treated mice.

**Figure 5:**
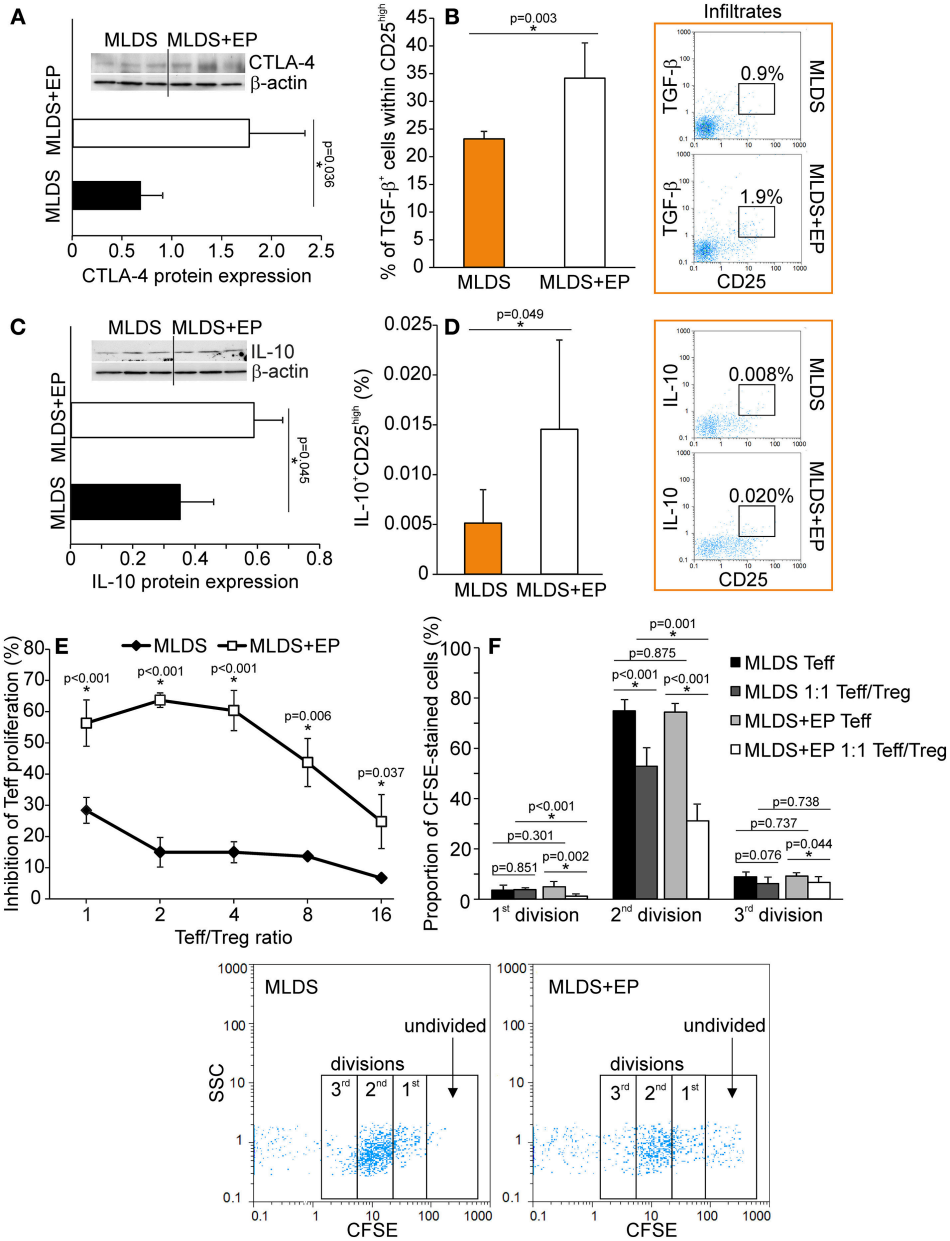
Functional characterization of Treg after EP treatment. **(A)** CTLA-4 protein expression in CD25^+^ cells purified from the pool of lymphoid tissues, normalized to the expression of β-actin, along with the representative blot. **(B)** The proportion of TGF-β-expressing cells within Treg, along with representative dot plots. **(C)** IL-10 protein expression in CD25^+^ cells purified from a pool of lymphoid tissues, normalized to the expression of β-actin, along with the representative blot. **(D)** The proportion of IL-10-expressing Treg within pancreatic infiltrates, along with representative dot plots. **(E)** The level of inhibition of effector T cell (Teff–CD4^+^CD25^−^) proliferation after co-culture with Treg (CD4^+^CD25^+^) cells purified from a pool of lymphoid tissues of MLDS or MLDS+EP-treated mice. Proliferation was measured after 72 h of incubation by CFSE dilution (in Teff). **(F)** Proportion of divided Teff, or Teff cultured in the presence of Treg (1:1 ratio). Representative dot plots are given below. All measurements were performed on samples from 7 animals per group. *p < 0.05 represents a statistically significant difference between cells of MLDS+EP compared to those of MLDS-treated mice.

To describe the phenotype of Treg in more detail, the expression of FoxP3 (signature transcription factor of Treg), GITR (glucocorticoid-induced tumor necrosis factor receptor, a marker of naturally occurring Treg) and CD127 (α subunit of IL-7 receptor that is absent on Treg) was determined within CD4^+^CD25^high^ cells. The results indicate that EP increased the number of CD4^+^CD25^high^FoxP3^+^ cells in PLN and pancreatic infiltrates (Figure 4A). Also, the proportion of CD4^+^CD25^high^CD127^−^GITR^+^ cells was increased after EP treatment (Figure 4B). The expression of Treg markers FoxP3, GITR, PD-1 per cell did not differ between the groups (Figure S5A), while the expression of cytotoxic T-lymphocyte antigen 4 (CTLA-4) in CD25^+^ cells from EP-treated mice was significantly increased (Figure 5A). EP's effect on their suppressive function was also manifested through a higher proportion of TGF-β^+^ Treg in EP-treated animals (Figure 5B), as well as through higher production of IL-10 in purified CD25^+^ cells (Figure 5C) and pancreatic infiltrates (Figure 5D). These data suggest that EP enhances Treg suppressive activity, which was confirmed in an *ex vivo* suppression assay (Figures 5E,F). Compared to CD25^+^ cells isolated from diabetic mice, cells from EP-treated mice were more potent in inhibition of CFSE-measured proliferation of CD4^+^CD25^−^ T effector cells. Overall, these results suggest that EP promoted Treg suppressive function through enhancing their capacity for effector T cell inhibition, both through cell-to-cell contact and secreted anti-inflammatory cytokines.

### *In vivo* EP Effect on Treg Differentiation and Proliferation

**Figure 6:**
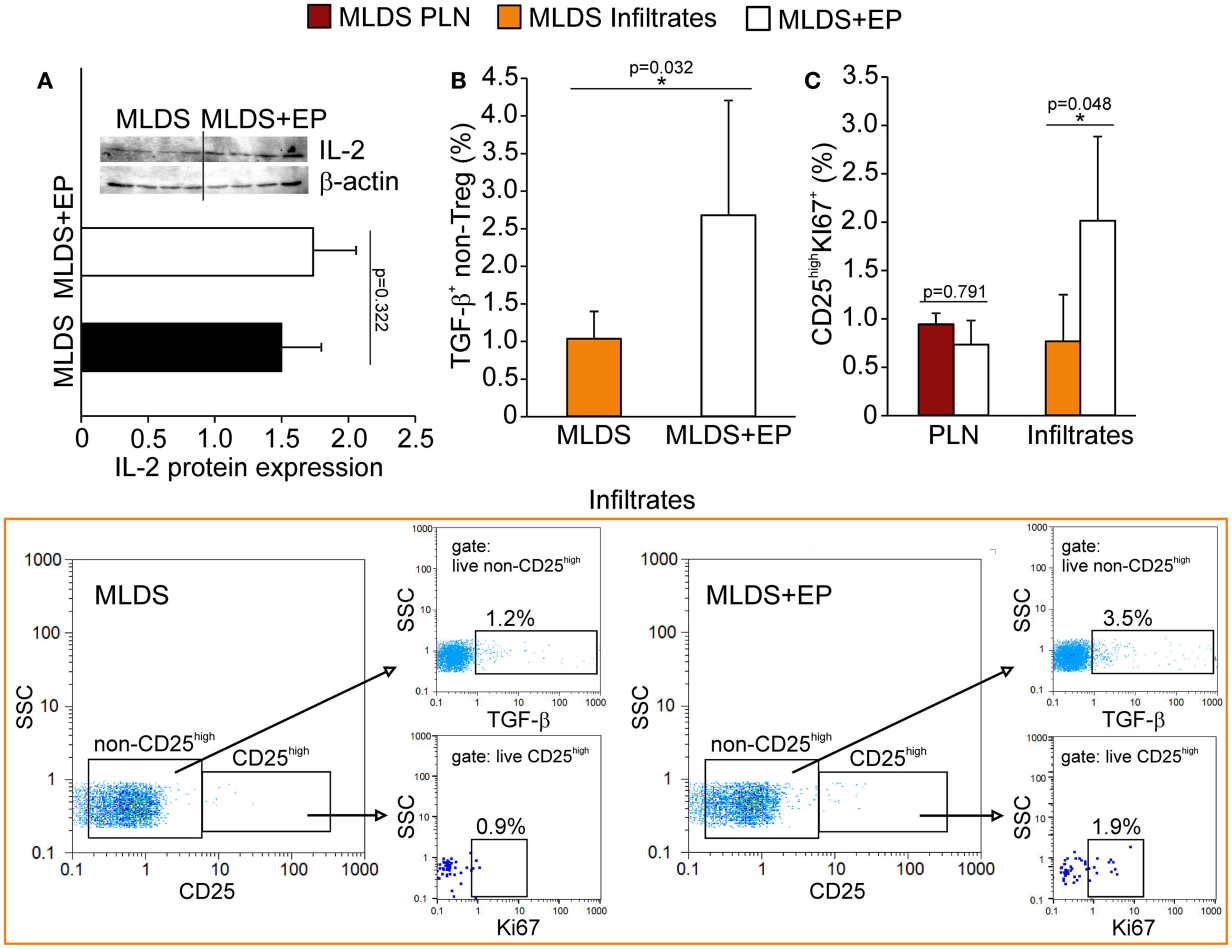
The effect of EP on Treg differentiation and proliferation *in vivo*. **(A)** IL-2 protein expression in PLN, along with the representative blot. **(B)** The proportion of non-Treg producers of TGF-β in the pancreatic infiltrates, along with representative dot plots (first gated on live non-CD25^high^ cells, followed by the gate on TGF-β^+^). **(C)** The proportion of proliferating Treg (CD25^high^Ki67^+^) in PLN and pancreatic infiltrates. Representative dot plots (first gated on live CD25^high^ cells, followed by the gate on Ki67^+^) for pancreatic infiltrates are shown. All measurements were performed on samples from 7 animals per group. *p < 0.05 represents statistically significant difference between cells of MLDS+EP compared to those of MLDS-treated mice.

The increased proportion of Treg found in both PLN and pancreatic infiltrates after EP treatment could be attributed either to increased *de novo* differentiation from naïve CD4^+^ cells or to the proliferation of existing ones. For peripheral differentiation of Treg, CD4^+^ cells require IL-2 and TGF-β in their surroundings (19). Although the presence of IL-2 in PLN stayed the same (Figure 6A), the proportion of TGF-β^+^ non-Treg cells was significantly elevated after EP administration (Figure 6B), suggesting that EP enhanced the TGF-β-mediated signaling pathway for enhanced Treg differentiation. However, it seemed that apart from differentiation, EP influenced Treg proliferation as well, judging by the increased number of Ki67^+^ Treg in the pancreatic infiltrates of EP-treated mice compared to those isolated from diabetic mice (Figure 6C).

### *In vivo* EP Effect on Treg Migration

**Figure 7:**
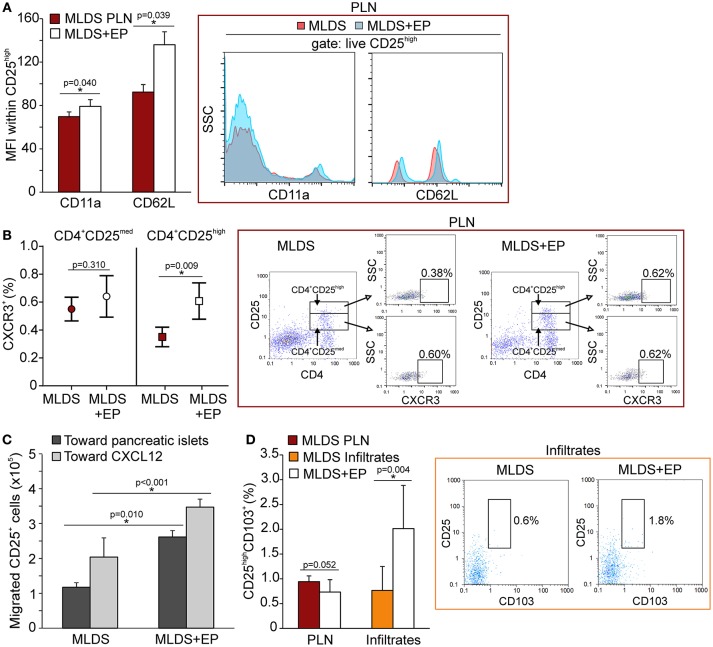
The effect of EP on Treg migratory abilities. **(A)** CD11a and CD62L expression on Treg within PLN, measured by mean fluorescence intensity (MFI). Representative histograms are shown. **(B)** The proportion of CXCR3^+^ cells within activated Th cells (CD4^+^CD25^med^) or within Treg (CD4^+^CD25^high^) from PLN. Representative dot plots show the first gate on either live CD4^+^CD25^med^ or live CD4^+^CD25^high^ cells, followed by the gate on CXCR3^+^. **(C)** Migration of CD25^+^ cells purified from a pool of lymphoid tissues of MLDS or MLDS+EP-treated mice in a chemotaxis assay toward pancreatic islets or CXCL12 (10 ng/ml). **(D)** CD25^high^CD103^+^ proportion within PLN and pancreatic infiltrates. Representative dot plots for pancreatic infiltrates are shown. All measurements were performed on samples from 7 animals per group. *p < 0.05 represents a statistically significant difference between cells of MLDS+EP compared to those of MLDS-treated mice.

Since the proportion of Treg was increased after EP treatment, the next step was to investigate their migratory capacities. In addition to the observed increased proliferation, the higher proportion of Treg might also result from their increased migration to the site of inflammation. We detected a higher expression of CD11a (subunit of LFA-1—integrin that drives cells from blood to tissues) and L-selectin (CD62L—adhesion molecule that enables transmigration) (Figure 7A) on Treg isolated from EP-treated mice, as well as an increased number of chemokine receptor CXCR3^+^ Treg (Figure 7B). The proportion of CXCR5^+^ Treg was the same between the groups (Figure S6A). These observations suggest that these cells migrated to the pancreas more readily in EP-treated mice than Treg from diabetic mice. This was confirmed in an *ex vivo* transmigration assay, in which CD25^+^ cells isolated from EP-treated mice migrated more efficiently toward pancreatic islets or toward a CXCL12 concentration gradient, compared to Treg isolated from diabetic mice (Figure 7C). Finally, EP increased the proportion of Treg that carried the CD103^+^ receptor in the pancreatic infiltrates (Figure 7D), while their proportion was similar in PLN of both diabetic and EP-treated mice (Figure 7D and Figure S6B) suggesting increased retention of Treg at the site of inflammation (20).

### The Effect of EP on Treg Activation and Affinity for Th1 or Th17 Suppression

**Figure 8:**
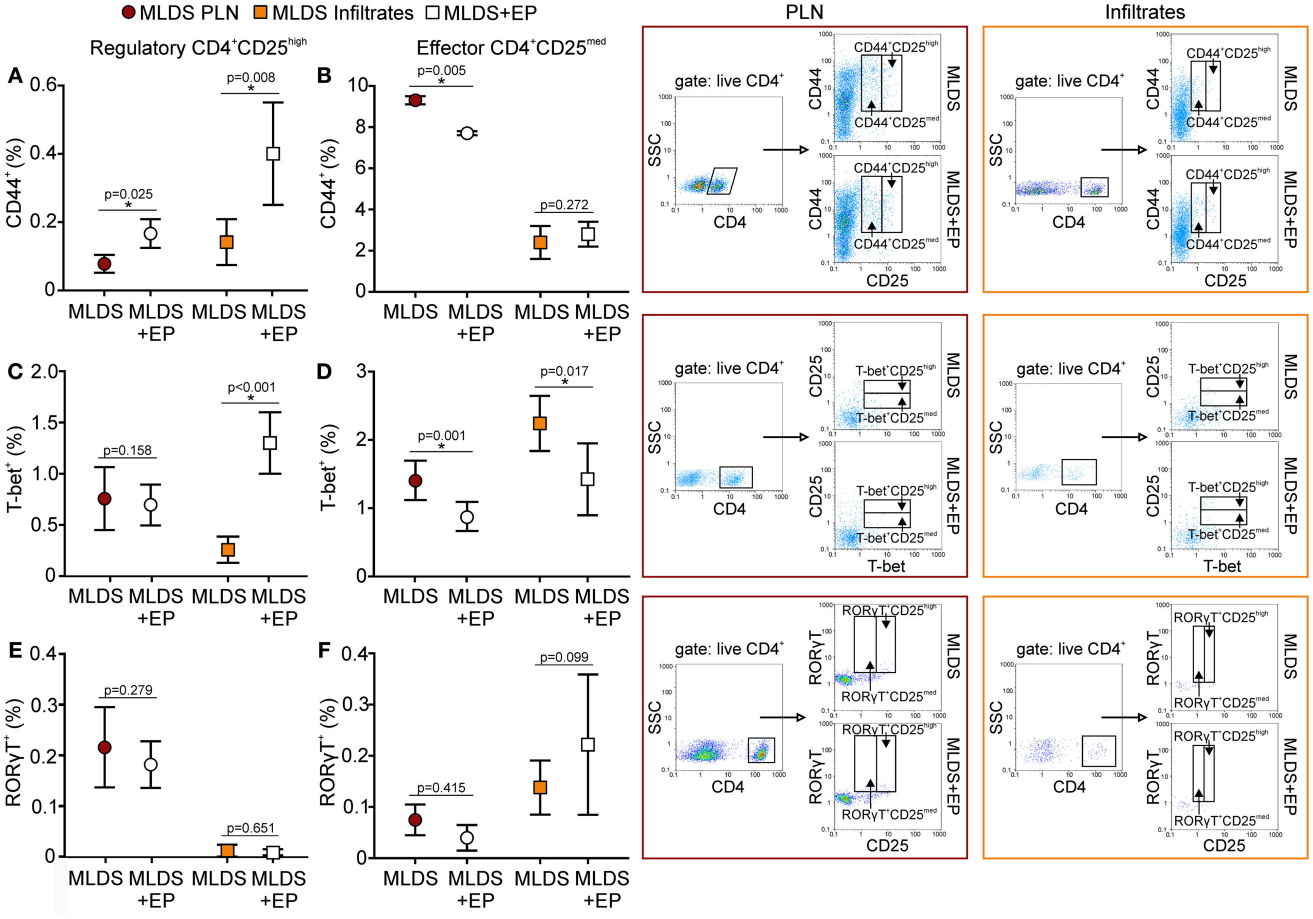
Activation and phenotype of Treg and effector T cells. The proportion of activated (CD44^+^), T-bet^+^, and RORγT^+^ cells within Treg (CD4^+^CD25^high^) **(A,C,E)** or within effector T population (CD4^+^CD25^med^) **(B,D,F)** was determined in PLN or infiltrates by flow cytometry. Representative dot plots are shown on the right hand side. Cells were first gated on live CD4^+^, followed by the gate on T-bet^+^ in CD4^+^CD25^med^ or CD4^+^CD25^high^ population. All measurements were performed on samples from 7 animals per group. *p < 0.05 represents a statistically significant difference between cells of MLDS+EP compared to those of MLDS-treated mice.

A higher proportion of Treg from EP-treated mice was CD44^+^, suggesting that they became activated (Figure 8A) and were able to inhibit the activation of effector Th cells (CD4^+^CD25^med^) (Figure 8B). Induced peripheral Treg might express transcription factors such as T-bet or RORγT and these cells are thought to specifically inhibit effector T cell population, Th1 and Th17, respectively. Seemingly, in the pancreatic infiltrates EP significantly up-regulated the T-bet^+^ Treg population (Figure 8C), while the proportion of effector T-bet^+^CD25^med^ cells was reduced (Figure 8D). However, the proportion of RORγT^+^ Treg and effector RORγT^+^CD25^med^ was similar in both groups (Figures 8E,F). The expression of T-bet or RORγT per cell did not differ upon EP treatment (data not shown).

### *In vitro* EP Effect on Treg Differentiation

**Figure 9:**
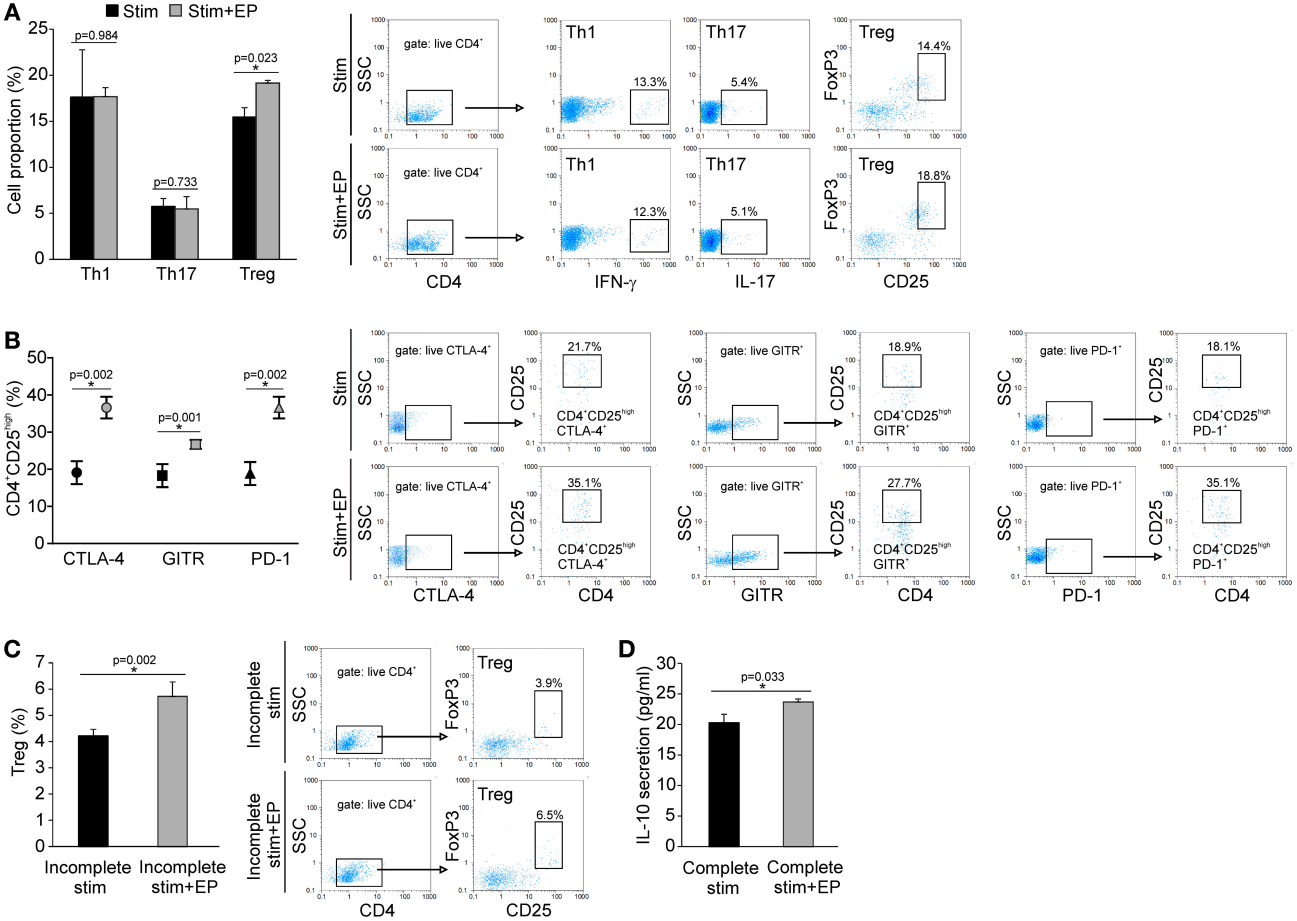
*In vitro* effect of EP on Th cell differentiation. **(A)** The proportion of Th1 (CD4^+^IFN-γ^+^), Th17 (CD4^+^IL-17^+^), and Treg (CD4^+^CD25^high^FoxP3^+^) cells after 96 h of incubation of CD4^+^CD25^−^ cells with adequate stimulation (described in Material and methods) and in the presence of EP (125 μM), added 24 h after the start of cell cultivation. Representative dot plots are shown. Cells were first gated on live CD4^+^, followed by the gate on IFN-γ^+^, IL-17^+^ or CD25^high^FoxpP3^+^. **(B)** The proportion of Treg that expressed GITR, CTLA-4, and PD-1, determined by flow cytometry. Representative dot plots show the first gate on live CTLA-4^+^, GITR^+^ or PD-1^+^ population, followed by the gate on CD4^+^CD25^high^. **(C)** The proportion of Treg after incomplete stimulation (anti-CD3+anti-CD28+IL-2) after 5 days of incubation of CD4^+^ cells in the presence or absence of EP. Representative dot plots show the first gate on live CD4^+^ cells, followed by the gate on CD25^high^FoxpP3^+^. **(D)** IL-10 concentration in supernatants of EP-treated CD4^+^ cells in the presence of complete Treg stimulation (anti-CD3/anti-CD28+IL-2+TGF-β), after 5 days of incubation, measured by ELISA. *p < 0.05 represents a statistically significant difference between cells of MLDS+EP compared to those of MLDS-treated mice.

Naïve CD4^+^CD25^−^ cells were stimulated *in vitro* through their TCR in the presence of adequate cytokines or antibodies that drive the differentiation of Treg, Th1 or Th17 cells. The addition of EP 24 h after the initial stimulation significantly promoted the differentiation of Treg, while the proportion of *de novo* differentiated Th1 and Th17 cells did not change compared to the cells cultured without EP (Figure 9A). The expanded Treg expressed PD-1, GITR and CTLA-4 surface markers (Figure 9B), but the levels of expression per cell did not differ compared to Treg developed without EP (data not shown). EP also stimulated Treg *in vitro* differentiation in the absence of TGF-β (incomplete stimulation) (Figure 9C), suggesting that EP might have stimulated, at least partly, the intracellular signaling events that mimicked the TGF-β trigger. Finally, EP stimulated IL-10 production in the CD4^+^ cultures exposed to the complete Treg stimulation (Figure 9D). These data suggest that EP stimulates differentiation of Treg in culture.

## Discussion

EP exerted specific effect on the regulatory arm of the immune response and thus prevented the development of T1D in C57BL/6 mice. It promoted tolDC within the pancreas and influenced Treg biology at multiple levels: through the stimulation of Treg proliferation and *de novo* differentiation, through the increase of CTLA-4, TGF-β, and IL-10 expression, making them more suppressive, and through favoring their migration to and retention at the site of inflammation.

The β-cell death in the investigated mouse model of T1D occurs through the induction of oxidative stress by the action of streptozotocin (21). Since EP possesses anti-oxidant properties, to avoid possible false results due to interference of EP with streptozotocin, the application of these two chemicals was at least 3 h apart. β-cells are extremely sensitive to oxidative damage since they have low levels of protective anti-oxidant enzymes (22). When endangered, β-cells express alarmins. One of those is HMGB1, whose level in β-cells of NOD mice correlates with the initiation of T1D (15). Higher HMGB1 expression was also found in β-cells of C57BL/6 mice treated with streptozotocin. EP is usually characterized as an HMGB1 inhibitor and it undoubtedly did reduce HMGB1 presence in pancreatic β-cells. Aside from acting as an alarmin, HMGB1 might be detrimental to β-cells because it can induce ROS generation through the stimulation of NADPH oxidase (4). Therefore, its reduction in β-cells may have helped protect β-cells from ROS-induced apoptosis. Surprisingly, EP did not prevent ROS and RNI generation in phagocytic immune cells that entered the pancreas, suggesting that this function of antigen-presenting cells is resistant to EP's influence.

After the initial ROS and RNI-mediated β-cell destruction after streptozotocin treatment, immune cells infiltrate the pancreatic islets. Subsequent β-cell apoptosis then becomes a result of a fulminant inflammatory process. In addition to anti-oxidant properties, EP can block inflammation as well. EP was found effective in several animal models of diseases with a strong inflammatory component, for example experimental colitis and encephalomyelitis (8, 10, 11). Although little is known about EP's molecular targets in immune cells, its anti-inflammatory properties are again related to the down-regulation of HMGB1 in non-immune cells. When secreted, HMGB1 can bind cell-surface receptors for advanced glycation end products (RAGE) and in that way intensify inflammation through generation or propagation of cells of innate and adaptive immunity. Another way of a pro-inflammatory HMGB1 effect could be the activation of TLR4. For example, elevated HMGB1 expression in β-cells of NOD mice is detected by the surface TLR4 (15). This activation of TLR4 can trigger NF-κB signal transduction, leading to the expression of pro-inflammatory cytokines and the up-regulation of leukocyte adhesion molecules, thereby promoting injury and inflammation (23, 24). Consistent with these literature data, the other reason for the preservation of β-cell function after EP treatment was a lower degree of immune cell infiltration into the pancreatic islets (as determined by the insulitis scoring). Although the composition of these infiltrates was more or less the same regarding both cells of the innate and adaptive immunity, two major differences between EP-treated and diabetic mice have been observed. One is the higher presence of tolDC, and the other is the increased proportion of Treg. It was already shown that secreted HMGB1 stimulates dendritic cell maturation and *in vivo* homing to lymph nodes (25). So, the observed stimulating effect of EP on the differentiation of tolDC might be conducted through the inhibition of HMGB1 secretion. This was also confirmed *in vitro* where EP contributed immensely to the differentiation of tolDC from bone marrow precursors (personal communication—unpublished data). The higher presence of tolDC in the pancreatic infiltrates may not be due to *de novo* differentiation of immature DC, as these cells may come from pancreatic lymph nodes where we detected a lower number of tolDC. Additionally, despite the fact that HMGB1 stimulates the differentiation of M1 macrophages, its inhibition by EP did not affect the ratio between M1 and M2 macrophages.

In the inflammatory setting during T1D progression, EP increased the number and the quality of Treg. The higher proportion of Treg could be a result of either enhanced proliferation or *de novo* differentiation. Proliferation of Treg was confirmed in the pancreatic islets, while for their differentiation we have only circumstantial evidence. For example, the level of TGF-β^+^ (non-Treg) cells was increased after EP treatment, suggesting that this TGF-β could drive new differentiation. Also, EP stimulated Treg differentiation *in vitro* in the circumstances of suboptimal stimulation (in the absence of TGF-β), suggesting that EP might trigger the same signaling pathways as TGF-β in naïve CD4^+^ lymphocytes. EP also enhanced the suppressive properties of Treg *in vivo*. It is already known that HMGB1 inhibits Treg function by down-regulating the costimulatory molecule CTLA-4, the Treg cell transcription factor Foxp3, and the Treg suppressor cytokine IL-10 (26). Therefore, our results with *in vitro* and *in vivo* EP application strongly correlate with the observed inhibition of HMGB1 *in vivo* after EP treatment. More precisely, EP increased the expression of CTLA-4, which through binding to CD80 and/or CD86 probably suppressed the activation of antigen-presenting cells (27). Also, EP increased the proportion of TGF-β^+^ and IL-10^+^ Treg and thereby significantly up-regulated their immunosuppressive properties, as shown by their potency in the inhibition of T effector cell proliferation. These Treg that proliferated in response to EP treatment were probably the so-called induced or peripheral Treg, due to their strict necessity for exogenous lipids and pyruvate which they utilize in the Krebs cycle to acquire energy [reviewed in 28]. Therefore, the addition of the stable form of pyruvate may have aided the differentiation and proliferation of peripheral Treg through a metabolic pathway as well.

Although the proportion of Treg was higher in both pancreatic lymph nodes and infiltrates examined after EP treatment, their migration to the pancreatic islets might be significantly increased if we take into account their *ex vivo* behavior in the transmigration assay. *Ex vivo* isolated Treg from EP-treated mice were attracted more efficiently to the pancreatic islets or to a CXCL12 chemokine gradient compared to the Treg from diabetic mice. Furthermore, they expressed higher levels of CXCR3, a chemokine receptor that responds to CXCL9, CXCL10, and CXCL11 and guides the cells to the site of inflammation (29), in this case the pancreatic islet where they are successfully retained (judged by CD103 expression). The expression of CXCR5 on CD25^high^ cells (presumably regulatory follicular T cells) was the same in both diabetic and EP-treated mice, suggesting that EP did not specifically direct Treg to the germinal centers of pancreatic lymph nodes where they can help the differentiation of Breg (30). This result was corroborated by equal levels of Breg found in lymph nodes and infiltrates in mice of both groups.

It is a general opinion that Treg express signature transcription factors of pro-inflammatory cell subsets, thereby increasing their specificity for suppressing those particular Th subsets. Treatment with EP increased the proportion of T-bet^+^ Treg (T-bet^+^CD25^high^) in the pancreatic infiltrates and simultaneously reduced the proportion of T-bet activated T cells (T-bet^+^CD25^med^). However, there is a discrepancy between this result and the equal levels of Th1 cells observed in diabetic and EP-treated mice. This might stem from the fact that CD8^+^ cells may express CD25 and T-bet as well 31. Although the inhibition of HMGB1 can theoretically interfere with Th17 polarization (32, 33), our results indicate that HMGB1 reduction by the action of EP did not result in the down-regulation of Th17 response, which was corroborated by the equal levels of both activated effector RORγT^+^CD25^med^ cells and RORγT^+^ Treg after EP treatment.

**Figure 10:**
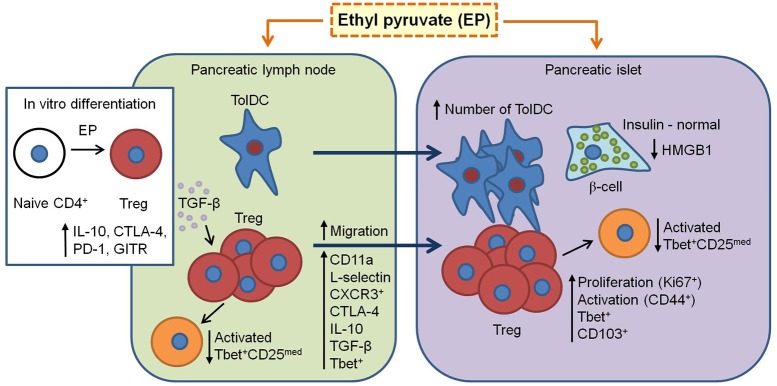
Proposed mechanism of EP's beneficial effect in T1D. EP enhances the regulatory arm of the immune response during T1D pathogenesis. From pancreatic lymph nodes, tolDC (CD11c^+^CD11b^−^CD103^+^) migrate into the pancreatic islets and their proportion becomes significantly higher. Also, Treg (CD4^+^CD25^high^) in the pancreatic lymph nodes increase their suppressive properties (CTLA-4, IL-10, and TGF-β) and migrate into the inflamed pancreatic islet (increased CD11a and CD62L expression and proportion of CXCR3^+^). In the islet, since Treg are already activated (CD44^+^), they proliferate (Ki67^+^), and are retained at the site (CD103^+^). Also, some of Treg express T-bet (increased proportion after EP treatment) and thereby inhibit T-bet^+^ effector cells (CD25^med^) within the PLN and pancreatic infiltrates. Finally, EP may stimulate *in vitro* and *in vivo* differentiation of Treg through enhancement of TGF-β production. All these events result in the preservation of β-cell function (insulin production) and reduction of HMGB1 expression.

In conclusion, this study proves the beneficial role of EP in the treatment of mouse T1D. It seems that EP favors the activation of the regulatory arm of the immune response and enhances its immunosuppressive properties (Figure 10). However, the question about EP's influence on the Treg metabolism still remains and should be explored as well.

## Author Contributions

All authors performed experimental work. IK and IS statistically analyzed the results. IS wrote the manuscript. All authors discussed data and reviewed the manuscript.

### Conflict of Interest Statement

The authors declare that the research was conducted in the absence of any commercial or financial relationships that could be construed as a potential conflict of interest.

## Supplementary Material

Figure S1 Negative staining for insulin **(A)** and HMGB1 **(B)**.

Figure S2 The effect of EP on innate cells proportion and function. **(A)** F4/80^+^ cell proportion within the spleen and the pancreatic infiltrates. Representative dot plots for the spleen are shown on the right hand side. **(B)** The proportion of M1 (F4/80^+^CD40^+^) and M2 (F4/80^+^CD206^+^) macrophages within the pancreatic infiltrates, with representative dot plots below. **(C)** The intracellular production of nitric oxide (NO) measured by DAF-FM mean fluorescence intensity (MFI) and reactive oxygen species (ROS) measured by DHR MFI within the pancreatic infiltrates, along with representative histograms. **(D)** The proportion of MHC class II^+^ antigen-presenting cells with co-stimulatory molecules CD80^+^ or CD86^+^ within pancreatic infiltrates, along with representative dot plots.

Figure S3 Phenotypic analysis of adaptive immune cells after EP treatment. Representative dot plots of the proportion of cytotoxic lymphocytes (CD8^+^) or B lymphocytes (B220^+^ or CD19^+^) in spleen **(A)**, PLN **(B)** or pancreatic infiltrates **(C)**. Representative dot plots of the proportion of regulatory B cells (CD19^+^CD5^+^IL-10^+^) within PLN **(D)** and pancreatic infiltrates (E) (first gated on live IL-10^+^ cells, followed by the gate on CD19^+^CD5^+^). **(F)** Representative dot plots of the proportion of activated cytotoxic lymphocytes (CD8^+^CD44^+^) in the pancreatic infiltrates.

Figure S4 Phenotypic analysis of adaptive immune cells after EP treatment. Representative dot plots of the proportion of Th (CD4^+^) and Th1 (CD4^+^IFN-γ^+^), Th2 (CD4^+^IL-4^+^) and Th17 (CD4^+^IL-17^+^) within the spleen **(A)**, PLN **(B)** and pancreatic infiltrates **(C)** of MLDS or MLDS+EP-treated mice (first gated on live CD4^+^ cells, followed by the gate on IFN–γ^+^, IL-4^+^, or IL-17^+^).

Figure S5 Characterization of Treg after EP treatment. **(A)** The expression of FoxP3, GITR, PD-1, and CD101 within CD4^+^CD25^high^ measured by mean fluorescence intensity (MFI), along with representative histograms.

Figure S6 The effect of EP on Treg migratory abilities. **(A)** The proportion of CXCR5^+^ cells within activated Th cells (CD4^+^CD25^med^) or within Treg (CD4^+^CD25^high^) from PLN. Representative dot plots show the first gate on either live CD4^+^CD25^med^ or live CD4^+^CD25^high^ cells, followed by the gate on CXCR5^+^. **(B)** Representative dot plots for CD25^high^CD103^+^ proportion within PLN.
