# Supplementary figures and images for: Ethyl Pyruvate Stimulates Regulatory T Cells and Ameliorates Type 1 Diabetes Development in Mice
Source: Front Immunol. 2019 Jan 10;9:3130. doi: 10.3389/fimmu.2018.03130 (PMC6335294; doi:10.3389/fimmu.2018.03130)

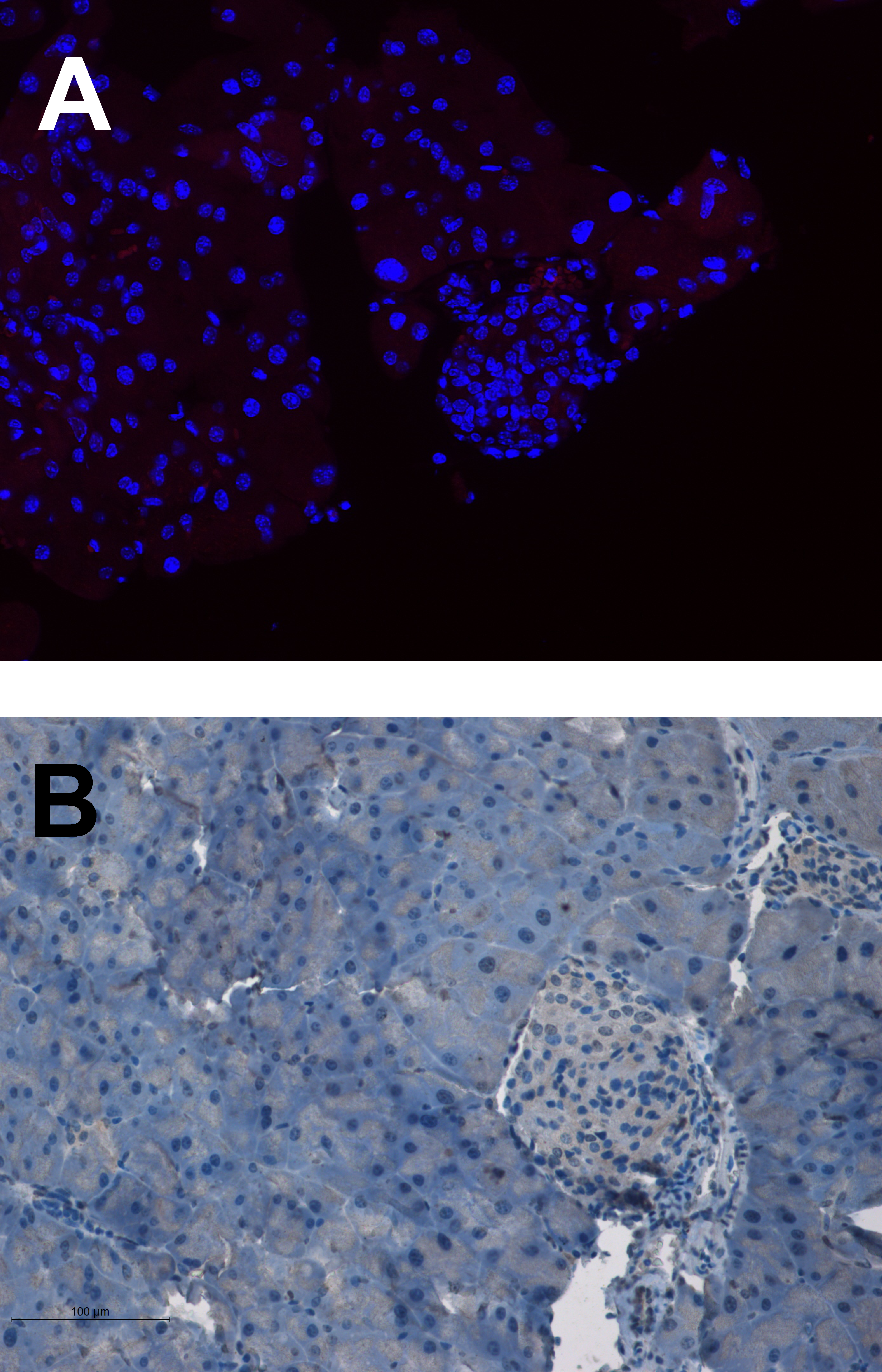

Supplement: Figure S1 — Negative staining for insulin (A) and HMGB1 (B). [file Image_1.TIF]

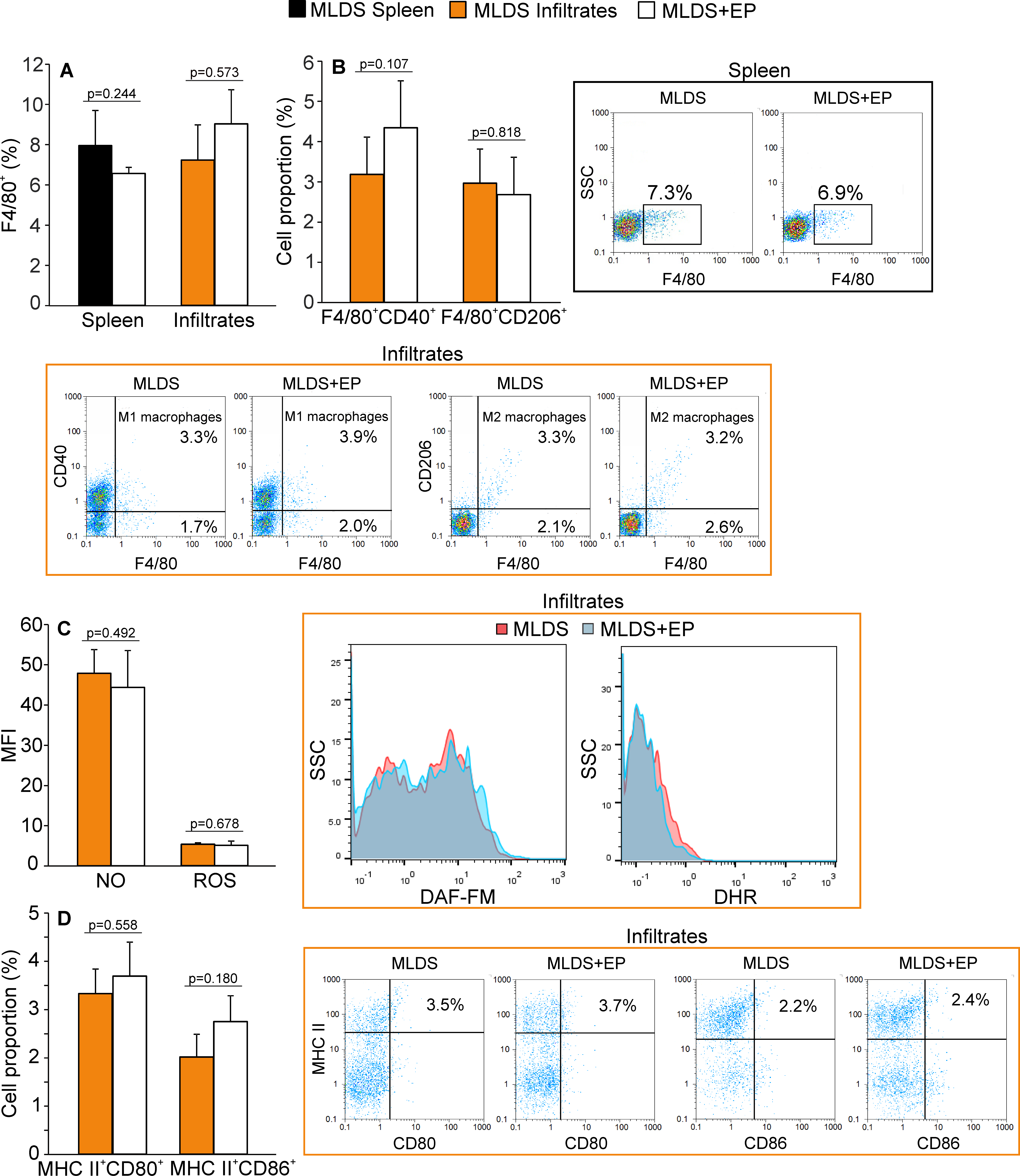

Supplement: Figure S2 — The effect of EP on innate cells proportion and function. (A) F4/80+ cell proportion within the spleen and the pancreatic infiltrates. Representative dot plots for the spleen are shown on the right hand side. (B) The proportion of M1 (F4/80+CD40+) and M2 (F4/80+CD206+) macrophages within the pancreatic infiltrates, with representative dot plots below. (C) The intracellular production of nitric oxide (NO) measured by DAF-FM mean fluorescence intensity (MFI) and reactive oxygen species (ROS) measured by DHR MFI within the pancreatic infiltrates, along with representative histograms. (D) The proportion of MHC class II+ antigen-presenting cells with co-stimulatory molecules CD80+ or CD86+ within pancreatic infiltrates, along with representative dot plots. [file Image_2.TIF]

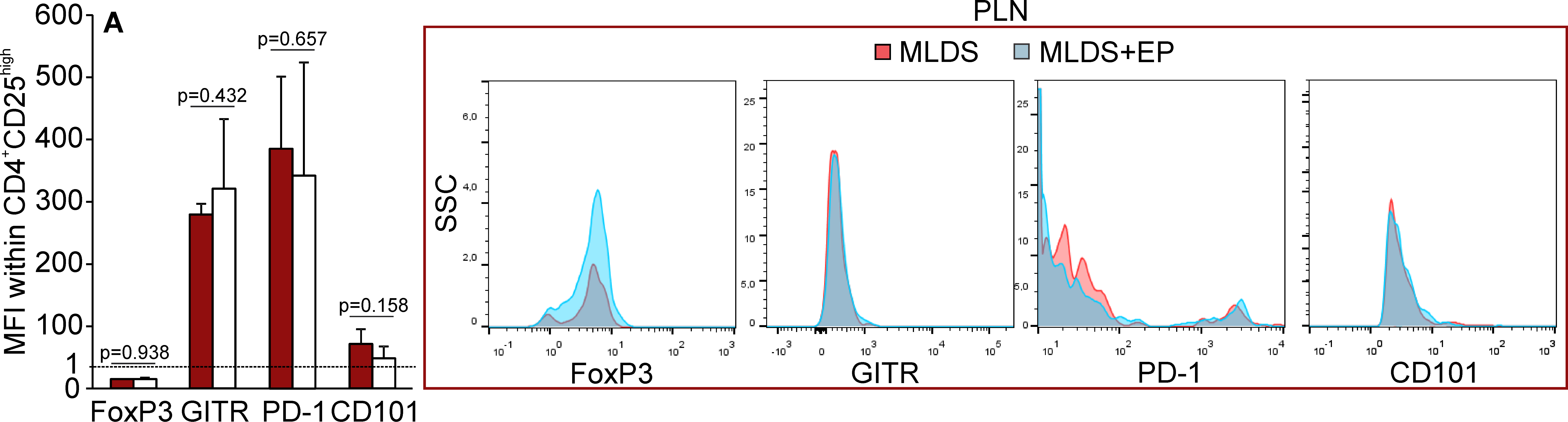

Supplement: Figure S5 — Characterization of Treg after EP treatment. (A) The expression of FoxP3, GITR, PD-1, and CD101 within CD4+CD25high measured by mean fluorescence intensity (MFI), along with representative histograms. [file Image_5.TIF]

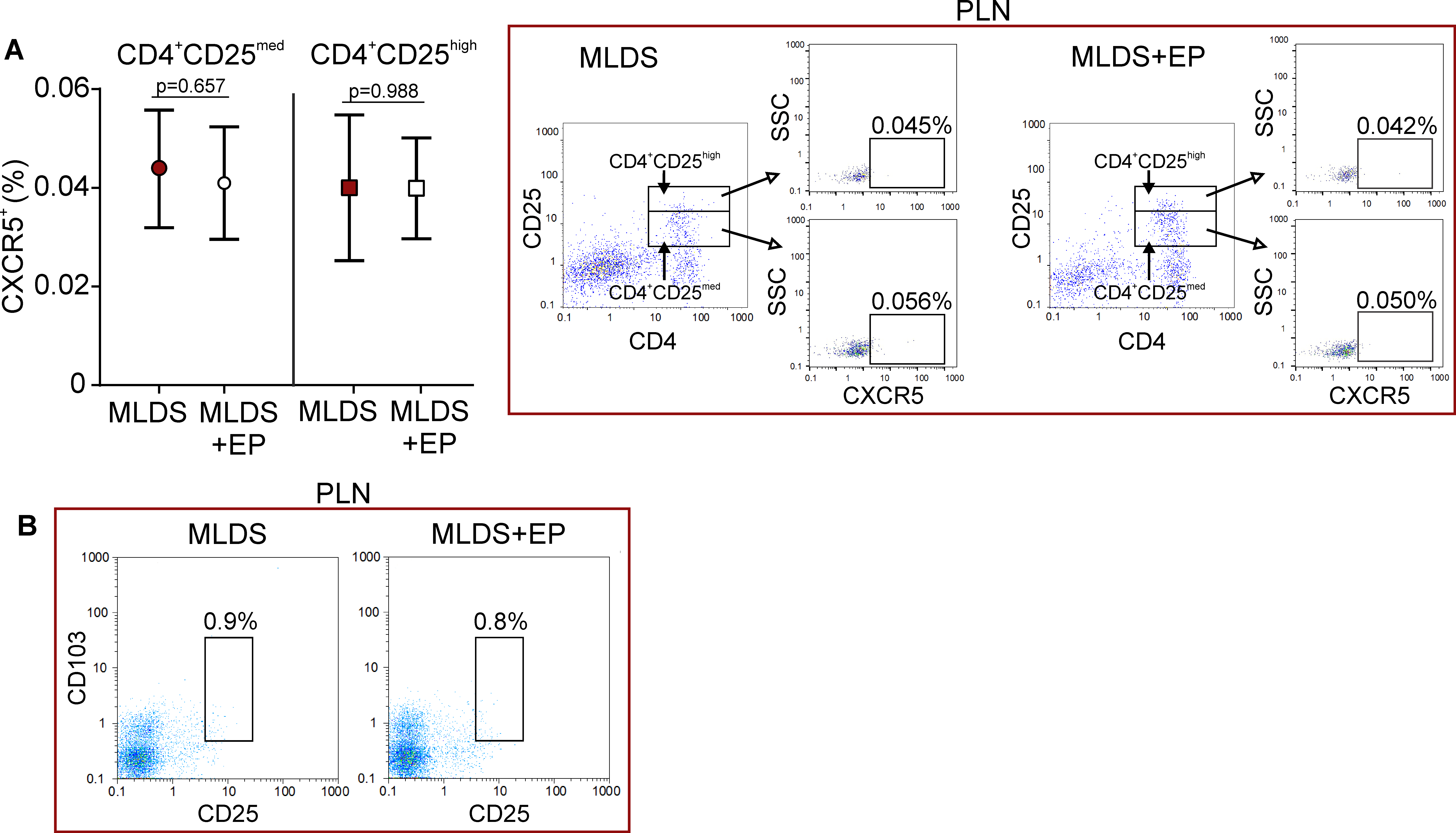

Supplement: Figure S6 — The effect of EP on Treg migratory abilities. (A) The proportion of CXCR5+ cells within activated Th cells (CD4+CD25med) or within Treg (CD4+CD25high) from PLN. Representative dot plots show the first gate on either live CD4+CD25med or live CD4+CD25high cells, followed by the gate on CXCR5+. (B) Representative dot plots for CD25highCD103+ proportion within PLN. [file Image_6.TIF]
